# A Cross-Country Study of Cigarette Affordability and Single-Stick Purchases Using Survey Data From African Countries

**DOI:** 10.1093/ntr/ntae097

**Published:** 2024-05-16

**Authors:** Samantha Filby

**Affiliations:** Research Unit on the Economics of Excisable Products (REEP), School of Economics, University of Cape Town, Cape Town, South Africa

## Abstract

**Introduction:**

Reducing cigarette affordability is paramount for reducing cigarette consumption. Measuring affordability requires data on cigarette prices. Unlike the commonly used retail price of a 20-pack of the most-sold cigarette domestically, survey-derived cigarette prices reflect differences arising from the brand variety and the types of packaging in which cigarettes are purchased.

**Aims and Methods:**

This paper uses self-reported cigarette price data from the eight African countries that have implemented the Global Adult Tobacco Survey (GATS) to construct country-level Relative Income Prices. The relationship between cigarette affordability, cigarette smoking prevalence, and cigarette smoking intensity, is examined using logit models for smoking participation (*N* = 51 122) and generalized linear models for conditional cigarette demand (*N* = 2443). GATS data are also used to produce nationally representative estimates of the prevalence of single-stick cigarette purchases in the sampled countries.

**Results:**

The estimated affordability elasticity of cigarette smoking participation is –0.245 (95% CI = –0.411 to –0.078). The estimated affordability elasticity of smoking intensity is –0.155 (95% CI = –0.286 to –0.023). Single-stick cigarette sales dominate all-markets. The proportion of smokers who reported buying cigarettes in the form of single sticks during their most recent cigarette purchase exceeds 90% in Kenya, Tanzania, and Uganda.

**Conclusions:**

The results point to the need for governments in the countries sampled to increase excise taxes in a manner that renders cigarettes less affordable over time, and to enact and enforce legislation that prohibits the sale of single cigarettes. These findings highlight that measures to reduce both the demand and supply of cigarettes will be required to reduce their use in the region.

**Implications:**

This study is the first to examine the prevalence of single-stick cigarette purchases, and the association between cigarette affordability and smoking outcomes, in the African setting, using data from the GATS. Findings provide local evidence for the countries sampled, which represent over half of sub-Saharan Africa’s adult population (aged 15 and older), on the importance of implementing excise tax increases that reduce cigarette affordability over time. They also highlight the need to enact and enforce legislation that prohibits the sale of single cigarettes.

## Introduction

Tobacco tax increases that raise prices reduce tobacco consumption.^1,2^ However, income growth can offset these tax and price increases, thereby limiting their impact on tobacco use.^3^ By implication, when incomes in a country are growing, the magnitude of the tax increases required to reduce tobacco consumption would be larger than the increases required under conditions of weak economic growth.^3^

The affordability of tobacco products, which is determined by the interplay of consumers’ income and tobacco prices, has therefore attracted increasing attention at the policy level.^4–6^ As a matter of best-practice, the Article 6 guidelines on the implementation of the World Health Organization Framework Convention on Tobacco Control (WHO FCTC) encourage countries to consider income dynamics when adjusting their tobacco tax rates, to ensure that tobacco products become less affordable over time.^7^

Matching the emphasis placed on the concept of affordability at the policy level, several multicountry studies have examined the association between cigarette affordability and cigarette consumption by estimating the affordability elasticity of demand.^3,8–11^ In this literature, the most widely used measure of cigarette affordability is the Relative Income Price (RIP).^3,8–11^ The RIP is commonly defined as the percentage of per capita GDP required to purchase 100 packs of 20 cigarettes (in total, 2000 sticks).^3,8–11^ The RIP, defined in this way, is also reported every 2 years by the WHO in its *Global Report on the Tobacco Epidemic* (GRTE).^12^

In a cross-sectional framework using data pooled from 28 high-income countries (HICs) and 42 low- and middle-income countries (LMICs) across 11 years (1990–2001), Blecher et al. (2004) estimate that a 1% increase in the RIP (and thus an equivalent decrease in the affordability of cigarettes) decreases per capita cigarette consumption by 0.53%.^8^ In a panel framework using data from 47 LMICs and 24 HICs for the period 2001–2014, He et al. (2018) estimate an affordability elasticity of cigarette consumption of −0.19, which implies that a 10% increase in the RIP is associated with a 1.9% decrease in per capita consumption.^9^ Using panel data from 124 LMICs, Nargis et al. (2021) estimate an affordability elasticity of –0.21.^10^ An analysis of cigarette affordability in ten Southeastern European countries of mixed income levels, seven of which are LMICs, estimates a RIP elasticity of –0.62.^11^

A common limitation acknowledged in this literature is the use of the retail price of a 20-pack of a single cigarette brand, such as the most-sold brand or the cheapest brand sold domestically, to construct RIP affordability indices.^8,9,11^ Kostova et al. (2014) provide a detailed account of the shortcomings associated with using the price of a single brand and a single packaging type to measure the true prices paid for cigarettes in a country.^13^

One limitation that they identify is that a single retail price cannot detect the variety of brands and prices that are available in each market.^13^ This distorts the average price in countries where a substantial number of smokers do not smoke the most-sold brand. Another limitation is that not all-smokers buy cigarettes in packs.^13^ Purchases of single-stick cigarettes are especially common in LMICs.^14–16^ Research also shows that the unit cost per cigarette differs depending on the type of packaging in which they are purchased.^15,17^ A high prevalence of single-stick purchases would therefore impact the overall price level in a country in a manner that is not reflected in the price of a 20-pack of cigarettes.^13^

Kostova et al. (2014) further note that single retail prices are often collected from one or two types of outlets and thus do not capture differences in prices paid across the multitude of outlets where individuals can purchase cigarettes.^13^ Moreover, if the single retail price is taken from producer price lists, rather than collected through observations at retail outlets, the results also do not capture price promotions and coupons.^18^ Another limitation of these prices is that they do not account for the fact that the average price level is affected by cigarette smoking intensity within a country.^13^ By the law of demand, heavier smokers are expected to buy cheaper cigarettes than low-intensity smokers.^13^

Taken together, Kostova et al. (2014) highlight that important sources of cross-country price variability are missed in analyses that rely on the retail price of a 20-pack of a single brand.^13^ To address these shortcomings, they advocate the use of prices reported in surveys to estimate aggregate prices at the country level.^13^ To illustrate their case, they used self-reported information provided by cigarette smokers in 15 countries on the amount that they spent on cigarettes, and the number of cigarettes that they bought of any packaging type, during their most recent cigarette purchase. These 15 countries, most of which are LMICs, had implemented a single wave of the Global Adult Tobacco Survey (GATS), in different years, at the time of Kostova et al.’s analysis.^13^

For each country, the authors derived “unit value” cigarette prices by dividing self-reported cigarette expenditure, during respondents’ most recent cigarette purchase, by the total number of cigarette sticks contained in that purchase.^13^ Each smoker was also assigned a weight that reflected their share of the country’s total cigarette consumption.^13^ The weighted median price paid for 2000 cigarettes was then divided by GDP per capita to produce country-specific RIPs. By design, these RIPs contain prices that reflect each country’s unique mix of individual consumption characteristics such as brand choice and preferred cigarette packaging. In a cross-country comparison of the derived prices and RIPs, the authors identified opportunities for improvements in the tobacco-tax policy of the sampled countries.^13^

With the first cross-country analysis of cigarette affordability using data from GATS, Kostova et al. (2014)^13^ made an important contribution by showing the efficacy of using these survey data to construct country-level cigarette prices and affordability indices.^19^ The analysis also contributed to our understanding of prices and affordability in LMICs, which is where most of the world’s current smokers live.^20^ However, at the time that Kostova et al. (2014) conducted their analysis, no African countries had implemented GATS.

A single wave of GATS has since been implemented in eight African countries—Botswana (2017), Cameroon (2013), Ethiopia (2016), Kenya (2014), Nigeria (2012), Senegal (2015), Uganda (2013), and Tanzania (2018).^21^ While researchers have used these African GATS data to understand the link between excise tax structure and cigarette price distributions,^22^ the price elasticity of cigarette demand,^23^ and inequalities in successful tobacco cessation,^24^ no study has used these price data to study the link between cigarette affordability and cigarette smoking. This paper aims to fill this gap.

While per capita cigarette consumption is the most-used measure of smoking behavior in the cross-country affordability literature,^8–11^ it is a stock value of both smoking participation and smoking intensity.^25,26^ A benefit of the individual-level data provided in GATS is that it permits a distinction between these smoking behaviors to be made. In the current study, the association between cigarette affordability, smoking participation, and smoking intensity is examined using Cragg’s two-part model of demand.^27^ Because deriving RIPs from GATS yields insight into the proportion of people who purchase cigarettes in the form of single sticks, nationally representative estimates of the prevalence of single-stick purchases are also reported in this study.

This research makes two key contributions. First, the analysis uses nationally representative GATS data to provide local evidence for African governments on the importance of increasing tobacco excise taxes in a manner that reduces cigarette affordability over time. Recent trends in cigarette affordability in the region show the need for this information to encourage tax policy change. According to data from the most recent GRTE, between 2020 and 2022, cigarettes became more affordable in 30 of the 42 African countries with RIP data available in both years and African countries have the lowest cigarette excise taxes of any region.^12^

Second, this paper provides nationally representative estimates of the proportion of people who purchase cigarettes in the form of single sticks. Research from other countries in the region has found that single cigarettes are widely sold in some countries on the continent.^14,15,17^ However, none have explored the prevalence of single-stick purchases using nationally representative data, and not all the countries included in the current analysis are covered in the existing studies.^14,15,17^Article 16 of the FCTC advises Parties to ban the sale of single cigarette sticks.^28^ Single-stick sales threaten the public health agenda because they are typically more accessible to youth and indigent individuals.^29^ They also limit individuals’ exposure to health warnings that usually appear on cigarette packs.^29^

Tobacco use is a preventable risk factor contributing to Africa’s growing noncommunicable disease burden.^30^ By exploring the issues of single-stick purchases and cigarette affordability in the sampled African countries, this study adds local evidence to a broader knowledge base that can encourage African governments to adopt evidence-based strategies to reduce tobacco use and its associated health and economic costs.

## Materials and Methods

The primary source of data used in this study is the Global Adult Tobacco Survey (GATS). GATS is a nationally representative, standardized survey of adults aged 15 and older that has been implemented in more than 30 countries.^31^ GATS collects individual-level information on respondents’ socio-demographic characteristics and exposure to a range of tobacco-control policies.^31^ Survey respondents, which include tobacco users and nonusers, are randomly selected through multistage, geographically clustered sampling methods.^31^ Each country-level dataset provides sampling weights that can be used to provide nationally representative estimates for adults aged 15 and older.^31^

Among cigarette smokers, GATS collects information that can be used to derive the average price paid for cigarettes in each country in a manner that reflects brand variety, the types of packaging in which cigarettes are purchased, and the different venues where cigarettes are bought. Specifically, GATS asks cigarette smokers to report the cigarette packaging type (eg single sticks, packs, and cartons), the number of individual cigarettes contained in each packaging type, and the number of each packaging type bought, during their most recent cigarette purchase.^31^ It also asks respondents to indicate the cost of this purchase.^31^ Several studies have used this information to estimate average and median prices paid for cigarettes in GATS-implementing countries on the basis of the derived unit values.^13,22–24,32,33^

As is the case for many countries that have implemented GATS, a drawback of the GATS data available for countries located in Africa is that they only provide information for a single year in each country.^21^ This prevents researchers from controlling for country-fixed effects that could influence the relationship between policy measures of interest and smoking outcomes and thus impedes the derivation of causal inferences from these data.^22,23,33^

However, analyses of the price elasticity of cigarette demand in a multiple-country setting using GATS, and other data from countries that only have a single year of data available, have demonstrated the viability of using pooled country cross-sectional frameworks to estimate the direction of the relationship between prices and smoking outcomes.^23,33–35^ In these frameworks, the association between prices and smoking outcomes is examined by relying on variations in prices, standardized to a common currency, across countries.^23,33–35^ Confounding biases from unobserved country differences that could affect the relationship between cigarette prices and cigarette smoking, such as social norms, are proxied by observable differences in countries’ incomes (eg gross domestic product (GDP) per capita), or the percentage of the population living below the poverty line, local rates of exposure to cigarette advertising and antismoking messaging.^23,33,34^

A prominent feature of studies that use survey data to produce price-elasticity estimates^1,2,26^ is the use of the two-part model of demand, developed by Cragg,^27^ to estimate the association between prices and smoking outcomes. The two-part model allows for the decision to smoke, and the decision of how much to smoke, to be modeled independently.^27^

In the first part of this model, which includes information from both smokers and nonsmokers, the probability of smoking participation is estimated.^1,26,27^ The second part of the model examines the number of cigarettes consumed by smokers (conditional cigarette demand) and only includes information provided by cigarette smokers.^1,26,27^ Cragg’s model is employed in the current study to estimate an affordability elasticity of smoking participation and an affordability elasticity of cigarette smoking intensity. The total affordability elasticity of cigarette demand is then calculated as the sum of the participation and conditional elasticities.^4,25^ This derived (total) elasticity is conceptually equivalent to that which is directly estimated in analyses that use aggregate data on per capita cigarette consumption.^4,25^ Researchers in the field have called for future studies to use survey data in their analysis of tax-based tobacco-control policies to permit the distinction between prevalence and intensity to be made.^36^

The variables used in the regression analysis are listed and defined in Table 1. These variables are consistent with the non-price controls used to study the factors associated with smoking in other studies that use GATS data, specifically,^23,33,37^ and the controls used in existing cross-country studies of affordability elasticities that rely on a broader range of data sources.^3,8–11^Appendix 1 provides details on how each of these variables was constructed based on the questions posed in GATS.

**Table 1.** Definition of Analysis Variables

Dependent variables		
Category	Variable	Definition
**Cigarette smoking outcomes**	Smoking participation	1 = respondent reported smoking cigarettes daily or less than daily, and 0 = did not report smoking cigarettes at all.
	Conditional demand	The average number of cigarettes smoked per day by current smokers.
**Independent variables**		
**Category**	**Variable**	**Definition**
**Tobacco control variables**	Relative Income Price of cigarette affordability	A country-level measure of the ratio of the median price paid for 2000 cigarettes to per capita gross domestic product. The higher the RIP, the less affordable cigarettes are, and vice versa.
	Local prevalence of cigarette advertising exposure	Percentage of respondents averaged at the primary sampling unit, who report having seen any advertisements or signs promoting cigarettes through any of the following channels in the last 30 d: television, radio, billboards, posters, newspapers, magazines, cinema, the Internet, public transportation vehicles or stations, public walls. This measure is included to account for country-specific characteristics that may influence smoking such as the local non-price tobacco-control environment.
	Local prevalence of antitobacco media messages	Percentage of respondents, averaged at the primary sampling unit, who report having seen any information about the dangers of using cigarettes, or any information that encourages quitting in newspapers, magazines, television, radio, or billboards, in the last 30 d. This measure is included to account for country-specific characteristics that may influence smoking such as the local non-price tobacco-control environment.
	POWE composite score	A country-level score ranging between 1 and 25 that proxies for countries’ implementation of non-price tobacco-control policies.
	Misinformed about the harms of tobacco smoking	1 = respondent does not know or believe that tobacco causes serious illness, and 0 = respondent does know or believe that tobacco causes serious illness.
**Socio-demographic variables**	Age	Respondent’s age in years.
	Age-squared	Respondent’s age in years, squared. Added to account for potential nonlinearity between age and smoking outcomes.
	Gender	1 = male, and 0 = female.
	Residence type	1 = urban, and 0 = rural.
	Highest level of educational attainment	1 = No formal education, 2 = Primary schooling completed, 3 = Secondary schooling completed, and 4 = Any form of tertiary education completed.
	Asset-based wealth quintile	1 = Lowest quintile, 2 = second-lowest quintile, 3 = third-lowest quintile, 4 = second-highest quintile, and 5 = highest quintile.
	Employment	1 = Employed, 2 = Unemployed, and 3 = Not in the workforce.
	Marital status	1 = Single/never married, 2 = Married/cohabiting and 3 = Divorced/Separated/Widowed.
	Proportion of the population living below the poverty line	Country-level variable measuring the percentage of the population living on less than $1.90 a day (at 2011 international prices), taken from the World Bank Development Indicators. This poverty line is set by the World Bank to classify people living in extreme poverty.

A description of the questions provided in GATS used to construct the variables listed in Table 1 is provided in Appendix 1 of the Supplementary File.

Consistent with the two-part framework^26,27^ and the empirical literature,^23,33,34,38–40^ models of smoking participation are estimated with a logit regression and the average marginal effects are reported. A generalized linear model (GLM) is employed to estimate the covariates of smoking intensity (part 2 of the model).^23,33,34,38–40^ As is standard practice, the number of cigarettes smoked by smokers enters the model in logarithmic form.^26,40^ Based on the most favorable Akaike’s Information Criterion Statistic, the GLM model is fitted with a Gaussian distribution and a log link.^41^ Average marginal effects and the affordability elasticities are estimated using Stata’s *margins* command^42^ and are reported at the mean characteristics of the sample. All-regressions were weighted to account for countries’ population size as detailed in Appendix 2.

Two versions of each model are run. In the primary analysis, the measures of local rates of exposure to cigarette advertising and antismoking messaging (Table 1), as provided in GATS, are used. A sensitivity analysis replaces these measures of the non-price tobacco-control environment with a composite score derived from the “POWE” components of the WHO MPOWER score (Appendix 1).

Respondents who did not report their smoking status (*n* = 619, 1.19% of observations) are excluded from the model. Missing data on the independent variables make up less than 0.3% of observations in the remaining datasets and are also excluded from the analysis. The final sample consists of 51 122 respondents from eight countries.

The analysis of the proportion of cigarette smokers who purchase cigarettes in the form of single sticks is descriptive and is based on self-reported information on the type of cigarette packaging purchased during respondents’ most recent cigarette purchase.

## Results

Descriptive statistics are shown in Table 2. Botswana has the highest cigarette smoking prevalence (12.7%), while Ethiopia has the lowest (2.8%). Among smokers, cigarette smoking intensity is lowest in Uganda (around 6 cigarettes/day) and highest in Ethiopia (around 10 cigarettes/day). Across all-countries, the majority of smokers purchase cigarettes in the form of single sticks. The proportion of smokers who purchased cigarettes in the form of single sticks during their most recent purchase is lowest in Ethiopia (60.7%) and exceeds 90% in Kenya, Tanzania, and Uganda (Table 2).

**Table 2.** Sample Characteristics

	Botswana	Cameroon	Ethiopia	Kenya	Nigeria	Senegal	Tanzania	Uganda
Number of observations^†^	4511	5238	9959	4372	9716	4256	4713	8357
**Individual-level variables: cigarette smoking**								
Current smokers (%)	12.69	5.75	2.79	7.05	3.69	4.17	5.58	4.23
Average number of cigarettes smoked per day by smokers	6.33	8.29	10.21	8.67	7.12	9.23	7.42	5.84
Smokers who bought cigarettes in the form of single sticks during their most recent cigarette purchase (%)	80.40	81.26	60.70	90.87	70.89	84.52	93.24	90.84
**Socio-demographic variables: smokers and nonsmokers**								
**Individual-level variables**								
Average age	36.59	33.58	31.23	33.64	33.65	34.55	34.53	33.98
Male (%)	48.05	48.31	49.94	48.78	50.04	48.53	47.86	47.20
Urban (%)	46.20	50.05	24.14	34.96	36.95	49.95	33.21	25.83
No formal education (%)	16.68	40.37	50.63	38.33	36.21	69.55	29.58	55.63
Population in the highest wealth quintile of all-eight countries combined (%)	52.21	18.66	6.45	9.13	24.63	25.54	15.22	3.74
Employed (%)	44.28	48.69	50.39	47.32	60.15	48.48	69.61	64.33
Single (%)	77.16	34.93	35.71	32.71	33.68	34.54	28.93	29.92
**Country-level variables**								
Living below the poverty line (%)	14.5	26.0	30.8	37.1	56.4	38.5	49.4	41.3
**Tobacco-control variables**								
**Individual-level variables**								
Misinformed about the harms of tobacco use (%)	4.73	4.63	12.02	7.22	17.65	6.13	7.71	5.42
**Primary Sampling Unit (PSU)-level variables**								
Average PSU-level advertising exposure rate (%)	21.43	80.17	3.55	18.67	20.86	40.06	34.59	24.63
Average PSU-level exposure rate to antitobacco messaging (%)	78.39	78.31	24.43	63.51	43.49	58.33	55.04	69.15
Number of PSUs	364	210	370	189	1058	244	204	400
**Country-level variables**								
Relative Income Price (%)	5.96	6.63	7.93	7.78	4.62	6.94	8.47	12.7
POWE composite score (out of 25)	14	17	16	17	13	19	14	11

Means and proportions derived using weights for complex survey design provided in each country’s GATS dataset. PSU stands for primary sampling unit.

^†^The number of observations reported corresponds to the number of complete observations used in the regression analysis. Sample sizes vary across the variables described in this table to allow the maximum number of non-missing observations to be utlilized.

In terms of their socio-demographic traits, the proportion of males and females in the full sample is evenly split (49.3% male, 50.7% female). The proportion of respondents living in urban areas is highest in Cameroon (50.1%) and lowest in Ethiopia (24.1%). In terms of respondents’ highest level of educational attainment, Senegal has the largest proportion of people with no formal education (69.6%), while Botswana has the lowest (16.7%).

Regarding factors that can be influenced by tobacco-control policy, the RIP is highest in Uganda (12.7%) and lowest in Nigeria (4.6%). The proportion of respondents who are misinformed about the harms of tobacco use is highest in Nigeria (17.7%) and lowest in Botswana (4.7%). Around 80% of respondents in Cameroon reported being exposed to cigarette advertising in the 30 days preceding the survey, while only 4% of respondents in Ethiopia reported such exposure. The highest rate of exposure to antismoking messages was reported by respondents in Botswana and Cameroon (78% in both countries), while the lowest was reported among respondents in Ethiopia (24%).

Results from the smoking participation and conditional demand models are presented in parts 1 and 2 of  Table 3, respectively.

**Table 3.** Regression Results From the Two-Part Model of Cigarette Demand

	Part 1	Part 2
	Smoking participation (logit: smoking = 1)	Conditional demand (Dep. Var. = ln(intensity))
	*N* = 51 122	*N* = 2443
**RIP**	–0.001^***^ (0.0005)	–0.020^**^ (0.009)
**Local rate of exposure to cigarette advertising**	0.005 (0.011)	0.118 (0.139)
**Local rate of exposure to antismoking messages**	0.002 (0.016)	0.038 (0.171)
**Misinformed about the harms of tobacco smoking (base = informed about the harms of tobacco smoking)**	0.026^***^ (0.009)	0.070 (0.130)
**Age**	0.008^***^ (0.001)	0.025^***^ (0.008)
**Age squared**	–0.0001^***^ (0.000)	–0.0003^***^ (0.0001)
**Male**	0.156^***^ (0.010)	0.235^*^ (0.121)
**Urban**	0.004 (0.005)	0.012 (0.044)
**Education (base = no formal education)**		
Primary schooling completed	0.003 (0.005)	–0.004 (0.055)
Secondary schooling completed	0.0002 (0.007)	–0.003 (0.070)
Any form of tertiary education	–0.012^**^ (0.006)	0.084 (0.097)
**Asset-based wealth (base = lowest wealth quintile)**		
Low	–0.012^**^ (0.006)	0.197^*^ (0.108)
Mid	–0.017^**^ (0.008)	0.240^**^ (0.113)
High	–0.026^**^ (0.010)	0.229^**^ (0.106)
Highest	–0.035^***^ (0.009)	0.222^**^ (0.092)
**Employment (base = employed)**		
Unemployed	0.008^**^ (0.003)	0.131^**^ (0.057)
Not in the workforce	–0.026^***^ (0.004)	–0.010 (0.074)
**Marital status (base = single/never married)**		
Married/cohabiting	–0.028^***^ (0.005)	0.025 (0.048)
Divorced/separated/widowed	0.017^***^ (0.004)	–0.005 (0.060)
**% of the population living below the PPP$1.90 poverty line**	–0.001^***^ (0.0003)	–0.004^***^ (0.002)
**Affordability elasticity**	–0.245^***^ [95% CI = –0.411 to –0.078]	–0.155^**^ [95% CI = –0.286 to –0.023]

Coefficients are average marginal effects. Standard errors are clustered by country and indicated in parentheses.

^*^ *p* < .1.

^**^ *p* < .05.

^***^ *p* < .01.

Lower cigarette affordability (ie a higher RIP) is significantly associated with lower cigarette smoking prevalence (*p* < .01) and lower smoking intensity (*p* < .05). The estimated affordability elasticity of smoking participation is –0.245. The estimated affordability of elasticity of smoking intensity is –0.155. Being uninformed about the harms of smoking increases the probability of smoking participation by 2.6% points (*p* < .01) (Table 3: part 1) but has no significant impact on smoking intensity (Table 3: part 2). Exposure to cigarette advertising and antismoking messages are not significant predictors of smoking participation or of smoking intensity. The coefficient on the “POWE” composite score is also statistically insignificant in both parts of the two-part model (Supplementary File: Appendix 3). This alternate model specification does not affect the sign or statistical significance of the tobacco-control variables shown in parts 1 and 2 of Table 3.

In terms of individual-level socio-demographic characteristics, being male is associated with a substantially higher probability of smoking (*p* < .01) and, to a lesser extent, a higher cigarette smoking intensity (*p* < .1). Both smoking participation and intensity increase as age increases, though the marginal effect is diminishing as age increases, as indicated by the negative coefficient on age-squared in both models.

Relative to respondents in the lowest wealth quintile, being in wealth quintiles 2–5 decreases the likelihood of smoking participation (Table 3: part 1). Conversely, being wealthier is associated with heavier smoking intensity (Table 3: part 2). The probability of smoking is lower by 1.2% points for respondents with any form of tertiary education compared to those with no formal schooling (*p* < .05) and is not significantly associated with smoking intensity. Compared to people who have never been married, the probability of smoking is 2.8% points lower among people who are married or cohabiting (*p* < .01). One’s relationship status has no statistical association with the number of cigarettes smoked per day (Table 3: part 2).

## Discussion

The results of this study show that both socio-demographic and policy-related factors influence the prevalence and intensity of cigarette smoking amongst adults in eight African countries. The main variable of interest—the RIP—is negatively and significantly associated with lower cigarette smoking prevalence and intensity. The total affordability elasticity of demand for countries in this sample, calculated as the sum of the participation and conditional elasticities,^4,25^ is –0.400. This estimate lies in the range found in existing cross-country research from LMICs.^3,8–11^

While the topic of cigarette affordability has been explored in samples of SSA countries,^15,43^ these investigations have not linked affordability to adult smoking prevalence or intensity. One study, from Ghana, examines the association between cigarette affordability and smoking initiation among adolescents.^43^ Using data on cigarette smoking from the Global Youth Tobacco Survey (GYTS), the authors estimate an affordability elasticity of smoking initiation of −0.490.^43^

The total affordability elasticity of cigarette demand estimated in the current study is lower than the price elasticity of cigarette demand found in previous research that uses GATS data from the same sample of countries (–0.495).^23^ The aforementioned study from Ghana^43^ similarly reports an affordability elasticity of smoking initiation that is lower than the price elasticity of smoking initiation found in parallel research^35^ that also uses GYTS data from Ghana. To varying degrees, lower affordability elasticity estimates than price elasticity estimates are also found in multicountry studies that concurrently estimate both parameters.^10,11^

This demonstrates a theoretical tenet of the affordability concept: the impact of tax-led price increases can be reduced by increases in people’s incomes.^3^ From a policy perspective, this indicates the importance of taking income into consideration when raising tobacco taxes to ensure that the affordability of tobacco is reduced. The results of the current study provide evidence, specifically for a sample of eight African countries, that less-affordable cigarettes are associated with less cigarette smoking.

The popularity of single cigarettes among cigarette smokers found in the current research echoes findings in the existing literature investigating this topic in the African region,^14,15,17^ though no nationally representative estimates had yet been produced. Article 16 of the FCTC requires that Parties ban the sale of single cigarettes.^28^ All-countries included in this analysis had ratified the FCTC at the time their GATS surveys were conducted, yet only Ethiopia, Kenya, and Nigeria had implemented legislation banning the sale of single cigarette sticks at this time.^20^ However, even in these three countries, more than half of cigarette smokers reported buying cigarettes in the form of single sticks. This suggests weak enforcement in these three countries.

Because they can facilitate access to cigarettes at a lower cost per purchase than for a full pack of cigarettes, and they prevent users from exposure to health warnings that typically appear on cigarette packs, single-stick sales undermine the public health agenda.^12,29^ The findings of the current study point to the need for governments in the countries sampled to enact and enforce legislation that prohibits the sale of single cigarettes.

This study is subject to limitations. GATS did not take place in all countries in the same year. There is therefore a risk of bias being created by the different periods of time. Furthermore, because GATS is a self-response survey, the data may be subject to reporting errors. The direction of the associated bias is unknown.

The framework for estimating the affordability elasticity of cigarette demand was also limited to single cross sections of data. Results therefore provide an indication of the direction of the relationship between cigarette affordability and smoking outcomes for the sampled countries. Scholars will be able to quantify this relationship more precisely as new waves of GATS are repeated.

In this future work, researchers should investigate the potential for developing individualized measures of the RIP, as has been done in studies focused on China,^44,45^ Bangladesh,^46^ and the United Kingdom,^47^ or sub-regional measures of affordability, as has been done for the Philippines.^19^ Although a strength of the current study was its use of aggregated prices that reflect the purchasing styles of smokers within countries, it was not possible to avoid reliance on GDP per capita as a proxy for personal income since GATS does not ask respondents about their personal income. However, there may be scope for matching individual-level or area-level income data from other surveys with the information contained in GATS. This opens the potential to use intra-country heterogeneity in RIP measures, based on both individual prices and individual incomes, to examine the association between affordability and smoking outcomes in the setting of African countries.

## Conclusions

This study provides governments in the sampled countries with evidence of the importance of increasing tobacco excise taxes in a manner that renders cigarettes less affordable over time to reduce the demand for them. Findings on the prevalence of single-stick cigarette sales during respondents’ most recent cigarette purchase, even among countries that have banned their sale, point to the need for governments to enact, and enforce, legislation that prohibits the sale of single cigarettes, in order to reduce the supply of cigarettes. Taken together, the findings of this study highlight that it will require a comprehensive tobacco-control strategy, one that includes measures to reduce both the demand and supply of cigarettes, to tackle their use in the region effectively.

## Supplementary material

Supplementary material is available at *Nicotine and Tobacco Research* online.

ntae097_suppl_Supplementary_Material

## Data Availability

All-data used in this study are publicly available at: https://www.cdc.gov/tobacco/global/gtss/index.htm
