# Supplementary material for: A Cross-Country Study of Cigarette Affordability and Single-Stick Purchases Using Survey Data From African Countries
Source: Nicotine Tob Res. 2024 May 16;27(4):611–9. doi: 10.1093/ntr/ntae097 (PMC11931211; doi:10.1093/ntr/ntae097)
Supplement: ntae097_suppl_Supplementary_Material [file ntae097_suppl_supplementary_material.pdf]

A cross-country study of cigarette affordability and single-stick purchases using survey data from African countries

Supplementary File

Appendix 1. Variable construction

This appendix provides detail on the questions posed in GATS to construct each of the variables employed in the analysis [1]. It also discusses the construction of the POWE composite score, which is derived from various years of the World Health *Global Report on the Tobacco Epidemic* (GRTE) [2-7]. The process of deriving these variables follows that employed in previous GATS-based research that estimates the two-part model of cigarette demand to determine price elasticities [8, 9].

1.1 Dependent variables

Two dependent variables are used in the analysis: cigarette smoking prevalence and cigarette smoking intensity.

1.1.1 Cigarette smoking participation

GATS asks all respondents “Do you currently smoke tobacco on a daily basis, less than daily, or not at all?” [1]. Respondents who indicate that they smoke tobacco daily or less than daily are then asked “On average, how many of the following products do you currently smoke each day/week?” [1]. Manufactured cigarettes are on this list of products. Respondents self-report the number of manufactured cigarettes that they smoke each day/each week [1].

In this study, smoking participation is defined by a binary indicator that is set equal to 1 if the respondent reported smoking cigarettes daily or less than daily, and set equal to 0 if they reported not smoking cigarettes at all.

It should be noted that while GATS collects information on whether respondents use any non-cigarette forms of tobacco, it does not collect information on the prices of these non-cigarette tobacco products [1]. Hence, only cigarette smoking is considered in the current study.

1.1.2 Cigarette smoking intensity

As mentioned, GATS asks respondents who indicated that they currently smoke tobacco products daily, or less than daily, to indicate**,** on average, how many manufactured cigarettes they currently smoke each day/week [1]. Respondents then self-report the number of cigarettes that they smoke each day/each week [1].

The information provided in relation to the number of cigarettes smoked each day/week is used to generate a variable that shows the average number of cigarettes smoked by cigarette smokers each day (cigarette smoking intensity). For daily smokers, no adjustments to the raw data are required since daily smokers reported the number of cigarettes that they currently smoke each day. For weekly smokers, the number of cigarettes smoked each day is derived by dividing the self-reported number of cigarettes smoked each week by 7 (the number of days in a week).

1.2 Independent variables

1.2.1 Tobacco control variables

(a) Relative Income Price of cigarette affordability

Two variables are required to construct this measure: cigarette prices and GDP per capita. Data on GDP per capita (in the year that each country implemented GATS) are obtained from the World Bank Development Indicators [10]. Data on cigarette prices are derived from GATS.

GATS asks daily smokers and less than daily smokers: "The last time you bought cigarettes for yourself, how many cigarettes did you buy?" [1]. Response options are: 'single cigarettes', 'packs', 'cartons', 'other (specify)' and 'never bought cigarettes' [1]. Respondents who select 'other' are asked to 'specify the other unit of cigarettes' [1].

For each of these packaging types, respondents are asked the number of each packaging type that they purchased the last time that they bought cigarettes for themselves. As an example, respondents who indicated that their last cigarette purchase took the form of packs of cigarettes are asked to indicate the number of packs that they bought [1]. Respondents who purchased cigarettes in the form of packs, cartons or an 'other' package type are also asked to indicate how many cigarettes were contained in each pack, or each carton, or each 'other' packaging type [1]. For respondents who purchased cigarettes in the form of single sticks, the reported number of single sticks purchased the last time they bought cigarettes is identical to the number cigarettes contained in each 'package type' .

The total number of individual cigarette sticks contained in each purchase was calculated by multiplying the number of each packaging type purchased by the total number of cigarette sticks contained in each packaging unit -.e.g. 4 packs of cigarettes containing 20 cigarette sticks

= 80 individual cigarettes and 10 single cigarettes = 1 single (as a cigarette package type) x 10 individual cigarettes = 10 cigarettes.

After providing the information required to calculate the number of individual cigarette sticks contained in their most recent cigarette purchase, respondents are then asked, “In total, how much did you pay for this purchase?” This amount is provided in local currency. Consistent with other research [8, 9, 11-14], for each cigarette smoker, the price per stick was calculated by dividing the reported purchase cost by the total number of cigarette sticks contained in each individual’s purchase.

To identify price outliers in the data in a manner consistent with previous research that uses African GATS data [14], derived per stick prices were multiplied by 20 to get the price per 20 cigarettes, a standard pack size, in local currency. These prices were converted into constant 2019 dollars using country-specific Consumer Price Indices obtained from the World Bank Development Indicators [10]. Constant prices were then transformed into a common dollar currency using country-specific purchasing power parity (PPP) conversion factors obtained from the World Bank Development Indicators [10].

Following previous research, to account for extreme outliers in the constructed price variable, any observation that is greater than thirty 2019 PPP dollars was dropped since these prices are unrealistically high and likely reflect data capture or reporting errors [14]. Subsequently, any observation greater than three standard deviations (SD) from the mean price in each country was dropped from the analysis [14].

Self-reported prices per 20 sticks were then multiplied by 100 since the price of 2000 cigarette sticks is used to estimate the Relative Income Price [15-19]. Consistent with the approach adopted by Kostova *et al*. [12], and other research [20], consumption weights, calculated as the ratio of each individual's consumption to the total consumption in the sample, were used in estimating the median price paid for 2000 cigarette sticks in each country. Following Kostova *et al*. [12], the weighted median price of 2000 cigarette sticks in each country was then divided by GDP per capita in the year of each country's survey expressed in constant 2019 PPP dollars to obtain country-specific Relative Income Prices.

(b) Local prevalence of cigarette advertising exposure

To account for country-specific characteristics that may influence smoking, both the model of smoking participation and the model of smoking intensity control for the local prevalence of cigarette advertising exposure [8, 9, 21-23]. GATS asks all respondents if, in the last 30 days, they had seen any advertisements or signs promoting cigarettes through any of the following channels: television, radio, billboards, posters, newspapers, magazines, cinema, the internet, public transportation vehicles or stations, public walls [1].

Consistent with previous research [8, 9, 21-23], this variable is constructed as the Primary Sampling Unit (PSU)[1]-level mean of a binary individual-level variable indicating whether the respondent had recently (in the last 30 days) seen any advertisements or signs promoting cigarettes in any of the aforementioned channels. Aggregating individual-level exposure at the PSU level prior to inclusion in the models of smoking behaviour reduces the reverse causality bias between individual smoking and exposure to smoking-related information is reduced and

[1] GATS uses a geographically clustered multistage sampling methodology to identify the specific households that Field Interviewers will contact. First, a country is divided into Primary Sampling Units, segments within these Primary Sampling Units, and households within the segments. Then, a random sample of households is selected to participate in GATS. See The GATS Analysis and Reporting Package for further detail.

helps make sure that the direction of the relationship flows from advertising exposure to smoking and not *vice versa*.

(c) Local prevalence of anti-tobacco media messages

To account for country-specific characteristics that may influence smoking, both models control for the local prevalence of exposure to anti-tobacco messaging [8, 9, 21-23]. GATS asks all respondents if, in the last 30 days, they had seen any information about the dangers of use or that encourages quitting of tobacco products in any of the following settings: television, radio, billboards, posters, newspapers, magazines, cinema, the internet, public transportation vehicles or stations, public walls [1].

Consistent with previous research, this variable is constructed as the PSU[2]-level mean of a binary individual-level variable indicating whether the respondent had recently (in the last 30 days) seen any information about the dangers of use or that encourages quitting of the following tobacco products through any of the aforementioned channels [8, 9, 21-23].

Aggregating individual-level exposure at the PSU level prior to inclusion in the models of smoking behaviour reduces the reverse causality bias between individual smoking and exposure to information that discourages smoking and helps make sure that the direction of the relationship flows from anti-smoking messaging to smoking and not *vice versa* [8, 9, 21-23].

(d) "POWE" Composite Score. In 2008, the World Health Organization introduced a measure to assess countries' implementation of the key demand-reduction measures recommended by the Framework Convention on Tobacco Control [24]. This measure is called

[2] Same as the note indicated in 1 above.

the MPOWER score. The MPOWER score assigns points to countries in each of the following areas :"**M**onitor tobacco use"; "**P**rotect people from tobacco smoke"; "**O**ffer help to quit tobacco use"; "**W**arn about the dangers of tobacco"; "**E**nforce bans on tobacco advertising, promotion and sponsorship"; and **R**aise taxes on tobacco products".

For the "M" policy dimension, the score values range from 1 to 4 in which a score of 1 represents "no known data or no recent data or data that are not both recent and representative", and a score of 2–4 represents the weakest to the strongest level of the policy [25]. For the other five policy elements (POWER), the score measures its overall strength on a scale of 1 to 5 in which a score of 1 represents " Data not reported" and a score of 2–5 represents the weakest to strongest level of implementation of these policies [25].

An MPOWER composite score is calculated by adding up the different score in each individual component of MPOWER. Importantly, for the "W" component of MPOWER, a country is scored twice: first on a scale of 1-5 for the health warnings of cigarette packages, and second on a scale of 1-5 for the mass media anti-tobacco campaigns. In both cases, a score of 1 represents "Data not reported" and a score of 2–5 represents the weakest to strongest level of implementation of these policies.

This means that a country can earn a maximum of 34 points. The scores range from 7 (1 in each of the seven MPOWER components: recall the W component is scored twice) and 34 (4 in M component and 5 in six POWER components: recall the W component is scored twice).

For the purpose of this paper, the "M" and "R "components of the MPOWER composite scores to obtain the "POWE" score for each country in the sample. This is because the "M" component

measures the extent to which countries "monitor tobacco use and prevention policies". This does not reflect the state of tobacco-control policy implementation in a given country. The "R" component of the MPOWER package is excluded because the regressions already control for cigarette prices.

To calculate the "POWE" composite score for each country, the different scores for each of the individual components of "POWE", bearing in mind that the "W" component is scored twice. This means that a country can earn a maximum of 25 points. The scores range from (1 in each of the five POWE components) and 25 (a score of five in each of the POWE components).

(e) Misinformation about harms of tobacco smoking. GATS asks all respondents: "Based on what you know or believe, does smoking tobacco cause serious illness?" This is modelled as a binary indicator equal to one if the respondent answered "No" or "Don't Know" to the question, indicating that the respondent does not believe/does not know that smoking tobacco causes illness. The indicator was set equal to zero if the respondent answered "Yes" to the question, indicating that they are informed about the health harms of tobacco use.

1.2.2 Socio-demographic variables

(a) Age. GATS asks the age of all individuals who are surveyed [1]. This is a continuous variable. In addition to controlling for age, age also enter the model in quadratic form (i.e., age and age squared appear together). This accounts for the fact that the relationship between age and smoking outcome may be non-linear. For example, in the smoking participation equation with age and age^2, if the coefficient on age is positive, and the coefficient on age^2 is negative, the quadratic has a parabolic shape. In this way, including age as a quadratic term allows exploration into the possibility that the relationship between age and the likelihood of smoking participation may change after a certain point.

(b) Gender. GATS asks all individuals to identify their gender as either male or female [1]. This is modelled as a binary indicator equal to one if the respondent is male and zero if the respondent is female.

(c) Residence type. Information on respondents' residence type is provided in the publicly available data files. This is a binary indicator equal to one if the respondent lives in an urban area, and zero if they live in a rural area.

(d) Education. GATS asks individuals to provide the highest level of education that they have completed [1]. The education systems differ across countries, which resulted in different education levels being reported across the different surveys. Therefore, a relative measure of educational attainment was created, in line with the approach adopted by Nargis *et al.* [26]. The relative education categories included in the analysis are "No formal education," "Primary schooling completed," "Secondary schooling completed," and "Any form of tertiary education."

(e) Asset-based wealth quintile. GATS does not collect data on personal or household income. Instead, respondents are asked about their possession of different household items [1]. Multiple correspondence analysis is used to construct an asset-based wealth index based on each respondent's built environment and ownership of private assets within a household [27]. Items included in the index are access to electricity and a flush toilet in the house, and whether anyone in the household has a fixed-line telephone, a mobile phone, television, radio, refrigerator, car, scooter or a washing machine [1]. Ownership of a computer, a bicycle or clock watch was not included across all country surveys. These items are therefore excluded from the wealth index.

The index itself is divided into five quintiles, with Quintile 1 being the poorest wealth category and Quintile 5 being the wealthiest category.

The allocation of a wealth quintile to a particular individual can be based on their wealth status in relation to others in their country, or in relation to all individuals from all eight countries. Consistent with previous research, each individual's wealth status was assessed in the context of the pooled cross section of data [8]. This approach to classifying an individuals' wealth status is more appropriate because it allows one to account for the fact that standards of living may vary greatly between countries. This ensures that each wealth quintile contains those individuals who are most similar in terms of wealth, despite their geography. It also avoids allocating people from different countries to the same wealth quintile despite that these people may live in countries that have vastly different incomes.

As an extreme example, consider two countries, Burundi and Qatar. In 2022, GDP per capita in Burundi was around 259 USD [10]. Qatar's GDP per capita was around 87 661 USD [10]. Being in the top income quintile in Burundi and being in the top income quintile in Qatar will clearly be two very different experiences. If one grouped the people in the top income quintile in Burundi together with the people in top income quintile in Qatar, one would be trying to liken people of vastly different incomes. By assigning people to a wealth category in a manner that accounts for the fact that standards of living may greatly between countries, one is able to compare people who are more similar in terms of their wealth status.

(f) Employment status. In GATS, survey respondents are asked to identify any one of the following as their main work status over the past 12 months: government employee; non-government employee; self-employed; student; homemaker; retired; unemployed but able to work; and unemployed but unable to work [1]. These options were then grouped into three

categories: (1) “Employed” (includes government employees, non-government employees, and those who are self-employed), (2) “Unemployed” (includes those who are unemployed, but able to work), (3) “Not in the workforce” (includes students, homemakers, those who are retired and those who are unemployed and unable to work).

(g) Marital status. GATS asks individuals their the marital status [1]. Respondents can identify as single, married/cohabiting, separated, divorced, or widowed [1]. These options were then classified into three categories: (1) “Single/never married”; (2) “Married/cohabiting” and (3) “Divorced/Separated/Widowed”.

(a) **Poverty headcount ratio at PPP$1.90 a day.**

Because there are no repeated cross-sections by country over time, the methodological tools with which to address unobserved country factors, such as social norms, that could influence the relationship between prices and smoking are limited. As argued in previous research, unobserved country differences can, to some extent, be proxied by observable differences in countries’ income [9], or the percentage of people living below the poverty line [8, 21]. Because the most readily available measure of income at the country level, GDP per capita, is already included in the RIP, to account for country-specific characteristics that may influence smoking, all models control the proportion of respondents living below the PPP$1.90 a day poverty line. These data were obtained from the World Bank Development Indicators [10].

Appendix 2. Weighting procedure for regressions

Given the pooled cross-sectional nature of the current study, consistent with previous research [8], to account for the fact that the GATS sample size "oversamples" some countries relative to the actual size of their adult populations, and "under-samples" others, the regressions employ weighted data. Total sample sizes for completed individual interviews in each of the countries included in the analysis, and the size of the population older than 15, are presented in Table S1.

Table S1. Sample sizes of African countries that have implemented GATS

Country	Survey Year	GATS sample (n)	Population aged 15 and older in millions (N)
Botswana	2017	4 643	1.45
Cameroon	2013	5 271	12.48
Ethiopia	2016	10 150	60.38
Kenya	2014	4 408	27.12
Nigeria	2012	9 765	93.33
Senegal	2015	4 347	8.24
Tanzania	2018	4 797	31.48
Uganda	2013	8 508	18.38

Weights for the regression were calculated as:

$$weight_i = \frac{\sum_{i=1}^{i=8} n_i}{n_i} \times \frac{N_i}{\sum_{i=1}^{i=8} N_i}$$

where n_i is the sample size of country i, and N_i is the size of the adult population in country i [8].

Appendix 3. Sensitivity analysis

This Appendix shows the results of re-running the regressions and replacing the local prevalence of cigarette advertising exposure and the local prevalence of anti-tobacco media messaging with the "POWE" composite scores for each country. The results of this exercise are presented in the two tables below.

Table S2.1 below provides the results of the original regression specification for smoking participation (left-hand column) with the results obtained by including the POWE composite score (the grey-shaded column).

Supplementary Table S2.1: Models of smoking participation under different specifications

	**ORIGINAL: PART 1 Smoking participation (Logit: Smoking = 1)**	**SENSITIVITY: PART 1 Smoking participation (Logit: Smoking = 1)**
	N= 51,122	N= 51,122
**RIP**	-0.001*** (0.0005)	-0.001*** (0.000)
**POWE Score**	—	-0.000 (0.001)
**Local rate of exposure to cigarette advertising**	0.005 (0.011)	—
**Local rate of exposure to antismoking messages**	0.002 (0.016)	—
**Misinformed about the harms of tobacco smoking (base = Informed about the harms of tobacco smoking)**	0.026*** (0.009)	0.024 (0.003)
**Age**	0.008*** (0.001)	0.008*** (0.000)
**Age squared**	-0.000*** (0.000)	-0.000*** (0.000)
**Male**	0.156*** (0.010)	0.156*** (0.004)
**Urban**	0.004 (0.005)	0.004* (0.002)
**Education (base = no formal education)**		
Primary schooling completed	0.003 (0.005)	0.003 (0.003)
Secondary schooling completed	0.000 (0.007)	-0.001 (0.004)
Any form of tertiary education	-0.012** (0.006)	-0.013*** (0.004)
**Asset-based wealth (Base = lowest wealth quintile)**		
Low	-0.012** (0.006)	-0.010*** (0.003)***

Mid	-0.017**<br>(0.008)	-0.017***<br>(0.003)
High	-0.026**<br>(0.010)	-0.026***<br>(0.003)
Highest	-0.035***<br>(0.009)	-0.033***<br>(0.004)
**Employment (Base = employed)**		
Unemployed	0.008**<br>(0.003)	0.009***<br>(0.004)
Not in the workforce	-0.026***<br>(0.004)	-0.027***<br>(0.004)
**Marital status (Base = single/never married)**		
Married/cohabiting	-0.028***<br>(0.005)	-0.031***<br>(0.003)
Divorced/Separated/Widowed	0.017***<br>(0.004)	0.015***<br>(0.004)
**% of the population living below the PPP$ 1.90 poverty line**	-0.001***<br>(0.000)	-0.001***<br>(0.000)
**Affordability elasticity**	-0.245***<br>[95% CI: -0.411 to -0.078]	-0.211***<br>[95% CI: -0.322 to -0.101]

Standard errors are clustered by country and indicated in parentheses.
**p* < .1; ***p* < .05; ****p* < .01.

The results of the sensitivity analysis for the model of conditional cigarette demand are presented in Table S2.2. The regression specification with the POWE composite scores is in the grey-shaded column, while the results of the original specification (which includes local rates of exposure to cigarette advertising and antismoking messages instead of the POWE composite scores) are in the unshaded column on the left.

Supplementary Table S2.2: Models of conditional cigarette demand under different specifications

	ORIGINAL PART 2<br>Conditional demand<br>(Dep. Var. = ln(intensity))	SENSITIVITY: PART 2<br>Conditional demand<br>(Dep. Var. = ln(intensity))
	N=2,443	N=2,443
**RIP**	-0.020**<br>(0.009)	-0.022**<br>(0.009)
**POWE Score**	—	-0.087<br>(0.060)
**Local rate of exposure to cigarette advertising**	0.118<br>(0.139)	—
**Local rate of exposure to antismoking messages**	0.038<br>(0.171)	—
**Misinformed about the harms of tobacco smoking (base = Informed about the harms of tobacco smoking)**	0.070<br>(0.130)	0.018<br>(0.057)
**Age**	0.025***<br>(0.008)	0.017**<br>(0.008)
**Age squared**	-0.000***<br>(0.000)	-0.000***<br>(0.000)
**Male**	0.235*<br>(0.121)	0.248***<br>(0.072)
**Urban**	0.012<br>(0.044)	0.036<br>(0.040)
**Education (base = no formal education)**		
Primary schooling completed	-0.004<br>(0.055)	-0.030<br>(0.042)
Secondary schooling completed	-0.003<br>(0.070)	-0.034<br>(0.067)
Any form of tertiary education	0.084<br>(0.097)	0.072<br>(0.073)
**Asset-based wealth (Base = lowest wealth quintile)**		
Low	0.197*<br>(0.108)	0.188***<br>(0.054)
Mid	0.240**<br>(0.113)	0.270***<br>(0.056)
High	0.229**<br>(0.106)	0.263***<br>(0.061)
Highest	0.222**<br>(0.092)	0.147**<br>(0.070)
**Employment (Base = employed)**		
Unemployed	0.131**<br>(0.057)	0.103*<br>(0.062)
Not in the workforce	-0.010<br>(0.074)	0.028<br>(0.070)
**Marital status (Base = single/never married)**		
Married/cohabiting	0.025<br>(0.048)	0.172**<br>(0.068)
Divorced/Separated/Widowed	-0.005<br>(0.060)	0.104*<br>(0.056)
**% of the population living below the PPP$ 1.90 poverty line**	-0.004***<br>(0.002)	-0.005***<br>(0.002)

**Affordability elasticity**	-0.155**<br>[95% CI: -0.286 to -0.023]	-0.166**<br>[95% CI: -0.298 to -0.033]

Standard errors are clustered by country and indicated in parentheses.
*p < .1; **p < .05; ***p < .01.

List of references

[1] World Health Organization. GATS questionnaire. Available: https://www.who.int/teams/noncommunicable-diseases/surveillance/systems-tools/global-adult-tobacco-survey/questionnaire [2023, December 6]. 2020.
[2] World Health Organization. WHO Report on the Global Tobacco Epidemic, 2015: raising taxes on tobacco. Luxembourg: WHO 2015.
[3] World Health Organization. WHO Report on the GlobalTobacco Epidemic, 2017: Monitoring tobacco use and prevention policies. 2017.
[4] World Health Organization. WHO report on the global tobacco epidemic 2019: offer help to quit tobacco use. 2019.
[5] World Health Organization. Global Report on the Tobacco Epidemic: Offer help to quit tobacco use. 2019.
[6] World Health Organization. WHO report on the global tobacco epidemic 2021: addressing new and emerging products. Available: https://www.who.int/publications/i/item/9789240032095. 2021.
[7] World Health Organization. WHO report on the global tobacco epidemic, 2023: protect people from tobacco smoke [online]. Available: https://www.who.int/publications/i/item/9789240077164 (accessed 17 September 2023). 2023.
[8] Filby S. Cigarette prices and smoking among adults in eight sub-Saharan African countries: evidence from the Global Adult Tobacco Survey. *Tobacco Control* 2022:tc-2022-057626.
[9] Kostova D, Tesche J, Perucic AM, *et al.* Exploring the relationship between cigarette prices and smoking among adults: a cross-country study of low- and middle-income nations. *Nicotine Tob Res* 2014;**16 Suppl 1**:S10-15.
[10] World Bank. World Bank Development Indicators. Available: https://databank.worldbank.org/source/world-development-indicators [2024, Feb 6]. 2024.
[11] Chaloupka FJ, Kostova D, Shang C. Cigarette excise tax structure and cigarette prices: evidence from the global adult tobacco survey and the U.S. National Adult Tobacco Survey. *Nicotine Tob Res* 2014;**16 Suppl 1**:S3-9.
[12] Kostova D, Chaloupka FJ, Yurekli A, *et al.* A cross-country study of cigarette prices and affordability: evidence from the Global Adult Tobacco Survey. *Tob Control* 2014;**23**(1):e3.
[13] Rossouw L, Filby S. Inequalities in successful tobacco cessation and tobacco cessation attempts: Evidence from eight Sub-Saharan African countries. *PLOS ONE* 2022;**17**(11):e0277702.
[14] Filby S, Walbeek CV, Pan L. Cigarette excise tax structure and cigarette prices in nine sub-Saharan African countries: evidence from the Global Adult Tobacco Survey. *Tobacco Control* 2022:tobaccocontrol-2022-057414.
[15] Blecher EH, van Walbeek CP. An international analysis of cigarette affordability. *Tob Control* 2004;**13**(4):339-346.
[16] He Y, Shang C, Chaloupka FJ. The association between cigarette affordability and consumption: An update. *PLOS ONE* 2018;**13**(12):e0200665.
[17] Nargis N, Stoklosa M, Shang C, *et al.* Price, Income, and Affordability as the Determinants of Tobacco Consumption: A Practitioner's Guide to Tobacco Taxation. *Nicotine Tob Res* 2021;**23**(1):40-47.
[18] Zubović J, Zdravković A, Jovanović O, *et al.* Affordability of cigarettes in ten Southeastern European countries between 2008 and 2019. *Tobacco Control* 2023:tc-2022-057716.

[19] Blecher EH. Affordability of Tobacco Products: The Case of Cigarettes. Available: https://tobacconomics.org/research/affordability-of-tobacco-products-the-case-of-cigarettes/ [2024, January 2]. 2020.
[20] Shang C, Chaloupka FJ, Zahra N, *et al.* The distribution of cigarette prices under different tax structures: findings from the International Tobacco Control Policy Evaluation (ITC) Project. *Tob Control* 2014;**23 Suppl 1**(0 1):i23-29.
[21] Filby S, van Walbeek C. Cigarette Prices and Smoking Among Youth in 16 African Countries: Evidence From the Global Youth Tobacco Survey. *Nicotine Tob Res* 2022;**24**(8):1218-1227.
[22] Kostova D, Ross H, Blecher E, *et al.* Is youth smoking responsive to cigarette prices? Evidence from low- and middle-income countries. *Tob Control* 2011;**20**(6):419-424.
[23] Nikaj S, Chaloupka FJ. The effect of prices on cigarette use among youths in the global youth tobacco survey. *Nicotine Tob Res* 2014;**16 Suppl 1**:S16-23.
[24] Hiilamo H, Glantz S. Global Implementation of Tobacco Demand Reduction Measures Specified in Framework Convention on Tobacco Control. *Nicotine & Tobacco Research* 2021;**24**(4):503-510.
[25] World Health Organization. WHO report on the global tobacco epidemic 2021: addressing new and emerging products. 2021.
[26] Nargis N, Yong H-H, Driezen P, *et al.* Socioeconomic patterns of smoking cessation behavior in low and middle-income countries: Emerging evidence from the Global Adult Tobacco Surveys and International Tobacco Control Surveys. *PLoS One* 2019;**14**(9):e0220223-e0220223.
[27] Stata Corp. Stata Manual: mca — Multiple and joint correspondence analysis. Available: https://www.stata.com/manuals/mvmca.pdf [2023, October 30]. 2023.
